# Managing, making sense of and finding meaning in advanced illness: a qualitative exploration of the coping and wellbeing experiences of patients with lung cancer

**DOI:** 10.1111/1467-9566.12601

**Published:** 2017-10-16

**Authors:** Emily Harrop, Simon Noble, Michelle Edwards, Stephanie Sivell, Barbara Moore, Annmarie Nelson

**Affiliations:** ^1^ Marie Curie Palliative Care Research Centre Division of Population Medicine School of Medicine, Cardiff University Cardiff UK; ^2^ Health and Care Research Wales Support Centre Cardiff UK

**Keywords:** Coping, Cancer, Qualitative, Wellbeing, sense of coherence, palliative care

## Abstract

Coping plays an essential role in maintaining the wellbeing of patients with cancer. A number of different coping responses and strategies have been identified in the literature. The value and relevance of meaning based coping theory has also been emphasised, including Antonovosky's Sense of Coherence (SoC) theory. Ten patients with advanced lung cancer were interviewed up to three times. A total of twenty in depth interviews were carried out, fully transcribed and data were analysed following a methodology of Interpretative Phenomenological Analysis. Three broad domains were identified to categorise the core life concerns of participants; making sense of and managing one's illness; maintaining daily life and relationships and confronting the future. Within these domains multiple coping themes are identified, which to varying degrees help to maintain patient wellbeing and quality of life. This article considers the relevance of SoC theory for understanding the coping experiences of patients with advanced cancer, and identifies resources and factors likely to support patient coping, with implications for health and social care services.

## Background

Lung cancer is the third most common cancer and the leading cause of cancer‐related death in the UK, with roughly two thirds of patients (67.9%) expected to die within a year of diagnosis (Cancer Research UK 2017). Lung cancer patients can experience a wide range of symptoms, including pain, fatigue, breathlessness and coughing, as well as side effects of chemotherapy such as altered sense of taste and nausea and vomiting (Kuo and Ma 2002, Molassiotis *et al*. 2011, Tishelman *et al*. 2007). The morbidity and mortality associated with cancer and its treatment also causes increased risk of distress and negative psychological outcomes (Link *et al*. 2005, Lin and Bauer Wu 2004, Molassiotis *et al*. 2011). These include confusion‐bewilderment, depression‐dejection, anxiety, intrusive thoughts, reduced concentration, feelings of irritation and boredom, frustration and disturbed sleep (Kuo and Ma 2002; Molassiotis *et al*. 2011).

Coping plays an essential part in maintaining the wellbeing of cancer patients (Link *et al*. 2005). Qualitative studies investigating the coping related experiences of cancer patients have described the importance of maintaining a sense of normality (Ellis *et al*. 2012, Lin and Bauer Wu 2004) and control over their illness and its effects (Bulsara *et al*. 2004). A variety of emotion‐focused coping strategies have been identified, including active and passive acceptance, maintaining hope and a positive attitude, denial, distraction and avoidance, as well as problem‐focused strategies such as accessing information and social support (Bulsara *et al*. 2004, Ellis et al 2012, Lin and Bauer Wu 2004, Link *et al*. 2005, Taleghani *et al*. 2006). Religious and spiritual approaches have also been identified including faith, praying, acceptance of disease as God's will and spiritual fighting (Taleghani *et al*. 2012, Lin and Bauer Wu 2004, Link *et al*. 2005).

Within some of this literature, the transactional ‘stress‐appraisal ‐coping’ framework (Lazarus and Folkman 1984) has been used to theorise patient coping and to distinguish between problem and emotion focused strategies commonly used by patients (e.g. Ellis *et al*. 2012, Kuo and Ma 2002, Link *et al*. 2005). Whilst useful for distinguishing between different types of coping strategies and situations, a need for ‘meaning based’ models is also identified, in particular for patients and families coping with advanced diseases (Milberg and Strang 2004; 2007). This is suggested in the growing evidence that positive psychological states, such as feelings of hope and meaning, are achievable and important for those living with advanced disease (Kylmä et al 2009, Lin and Bauer Wu 2004, Milberg and Strang 2007, Olsman *et al*. 2014). One such model which is suggested to be useful for conceptualising how patients with advanced diseases cope with and adjust to their illness is Antonovosky's (1979) salutogenic approach and Theory of Sense of Coherence (SOC) (Kvåle and Synnes 2013, Milberg and Strang 2007). Salutogenic (as opposed to pathogenic) approaches focus on how, in the face of adversity, people are able to stay healthy and maintain wellbeing. Key to this, is how people ‘bring order out of chaos’ and are able to make the world coherent to them. SOC has three core elements; Comprehensibility, Manageability and Meaningfulness. Comprehensibility refers to how people see the world and their ability to understand what happens around them. Meaningfulness refers to the way in which the person finds meaning in the situation and sees the demands as challenges worthy of emotional investment, whilst manageability describes the extent to which they are able to respond to the situation and that resources are available to meet the demands (Antonovosky 1979). The resources which support SoC have been described by Antonovsky (1987) as General Resistance Resources and include; material resources, knowledge, identity, social support, cultural stability, religion/philosophy.

Research in healthcare, which has typically used Antonovsky's ‘orientation to life questionnaire’, suggests that a strong SOC acts as a health promoting resource and is positively related to quality of life (Eriksson and Lindstrom 2006, 2007), with similar findings reported in studies of cancer patients (Kvåle and Synnes 2013). Qualitative researchers have also applied the model to; theorise coping amongst patients with brain tumours (Strang and Strang 2001); interpret the experiences of the next of kin of cancer patients receiving palliative home care (Milberg and Strang 2003, 2004); and consider the role of health professionals in promoting SoC amongst patients (Kvåle and Synnes 2013) and family carers (Milberg and Strang 2007). These studies have found manageability to be supported by; active information‐seeking strategies (Kvåle and Synnes 2013, Milberg and Strang 2004; 2007, Strang and Strang 2001); open communication and social support, sense of control, belief in one's self (Milberg and Strang 2004; 2007, Strang and Strang 2001); and coping mechanisms involving positive reappraisal, rationalisation, bargaining, distancing, humour and repression (Kvåle and Synnes 2013, Strang and Strang 2001). Comprehensibility was also found to be supported by information seeking and accessing the right level of information (Kvåle and Synnes 2013, Milberg and Strang 2004, Strang and Strang 2001), by patients own thoughts and theories on their illnesses and coping mechanisms of rationalisation and redefinition (Strang and Strang 2001). Meaningfulness was identified to be central for quality of life and to be created by close relationships, faith, work and hobbies, and enabled by reappraisal of life, finding reasons to live for, hope and feelings of accomplishment and self‐transcendence (Kvåle and Synnes 2013, Milberg and Strang 2003; 2007, Strang and Strang 2001). A literature review by Lin and Bauer Wu (2004) similarly identified six essential components of psycho‐spiritual wellbeing in terminally ill patients; prognostic/self‐awareness, effective coping, relationships and connectedness with others, sense of faith, autonomy, and living with meaning and hope.

In this article we present the findings from the QualFRAG study. This qualitative study explored the experiences of patients with advanced lung cancer who were participating in a large multi‐centre clinical trial. In terms of disease status, 95 per cent of patients who entered the main trial had advanced (stage 3 or 4) lung cancer, 58 per cent of participants died within 12 months, and only 18 per cent lived beyond two years (see Macbeth *et al*. 2015). The independent sub study reported here investigated patients’ experiences of participating in the clinical trial, their symptom burden and management and the impacts of their illness on their quality of life and daily living. Participant experiences of participating in the trial are reported elsewhere (Harrop *et al*. 2016). In this article, we discuss data on how patients made sense of, managed and coped with their illness and in the discussion section interpret our results with reference to SoC theory. Although more commonly investigated using quantitative approaches (see Eriksson and Lindstrom 2006, 2007), we decided to retrospectively apply this theory to our data due to its explanatory ‘fit’ with many of our own coping related themes, as well as those of other qualitative studies also using this model (e.g. Kvåle and Synnes 2013, Milberg and Strang 2003, 2007, Strang and Strang 2001).

## Methods

Following ethical approval from the local NHS Research Ethics Committee, a total of ten participants were recruited from three sites which were participating in the trial. All eligible patients were approached by their trial research nurse between September 2011 and December 2011, when the main trial was closed to recruitment. With the exception of two patients, all eligible patients who were approached entered into the study. We aimed to interview participants three times to better understand their perspectives as their disease progressed:

1. within 18 weeks of joining the trial;
2. 6 weeks after the first interview; and
3. 6 to 8 weeks after the second interview.

In total, 20 interviews were completed, with four patients completing all three interviews. With the exception of one patient (Brian) who withdrew due to his disappointment at being allocated to the control arm, all other attrition was due to deterioration or death. Key characteristics for patients in this sub study are detailed in Table 1. Information on participant age was not directly collected at the time of interviews, although was revealed in some transcripts. For participants whose age remains unknown, an estimated age range is given instead.

**Table 1:** Participant characteristics

Pseudonym	Gender	Age/ estimated age	Number of interviews
Mary	F	40s	3
Brian	M	75	1
Peter	M	65	3
David	M	80	1
Mark	M	70	1
Toby	M	60s	1
Ben	M	50s	2
Tom	M	50s	2
Jim	M	76	3
Paul	M	77	3
Total interviews			20

With the exception of one patient who was interviewed in clinic, all other patients were interviewed at home. Several patients had companions present during the interviews, although regrettably consent was not taken to use their data. The researcher took written informed consent before the start of the first interview. Interviews were carried out by two female researchers (EH, ME) who were previously unknown to participants. They did not have clinical backgrounds, although had some prior experience of interviewing patients or family members affected by advanced illness. Interviews lasted between 15 minutes and an hour and covered motivations for joining the trial, understandings of trial processes and experiences of symptoms, side effects and quality of life (see Appendix for summary of topics). Following each round of interviews, the interview schedules were adjusted to reflect the participant‐led content from the previous round. Interviews were digitally recorded, transcribed verbatim and fully anonymised.

The analytic framework for this qualitative sub study was based on Interpretative Phenomenological Analysis (IPA; Smith and Osborn 2003). IPA was chosen because its idiographic approach enables a more in depth exploration of the lived experiences of individual participants than in other types of thematic analysis, and also aims to understand the meaning that events have for participants. It is interpretative in the sense that the researcher's perspectives are also recognised (Smith and Osborn 2003). It begins with a single case as a basis to develop more general categories developed in a detailed case‐by‐case analysis. Following preliminary reading and annotation of the transcript, initial comments are grouped into themes. Connections between themes are developed until a thematic account of the case is achieved and connections across cases are then noted to identify group level themes (Smith and Osborn 2003). Two researchers (EH, ME) analysed the transcripts for themes following this methodology. A coding framework for group themes was developed and results were verified by the research team by independent review of a selection of transcripts.

## Findings

Three broad thematic domains were identified to describe the core life concerns and challenges experienced by participants; making sense of and managing one's illness; maintaining daily life and relationships and confronting the future. A number of coping related themes are described under each of these three headings.

### Making sense of and managing one's illness

#### (Mis)understanding and (non)acceptance of diagnosis

Trying to make sense of and understand a diagnosis of terminal lung cancer poses emotional, psychological and cognitive challenges for patients. Participants were not directly asked about their experience of being diagnosed with cancer, although this did feature in a number of interviews. Some patients spoke little about this aspect of their illness, but seemed to have understood and accepted the existence of the illness, if not necessarily the prognostic implications. Several other patients, however, clearly struggled to make sense of the clinical information in the context of their own lived experiences and low symptom burdens, which made it difficult for them to understand and accept that they were seriously ill. Despite reporting feeling well and able to live a normal life, at the time of his third interview Peter had been informed that that there was no more that could be done and that he could deteriorate quickly, leaving him in a state of distress and disbelief. David, who deteriorated rapidly following the first interview and was not interviewed again, similarly gave a detailed description of his daily routine to demonstrate how normal his functionality and life continued to be.

I go shopping, I go shopping everyday don't I? … what I done now, I'll tell you what I done this morning, I go, I went to church, uh I usually take (names wife) to church on Wednesday … then before I went to church I went to the butchers and ordered the meat. [David, interview 1]

By giving this account of the trivia of normal life, he seemed to be attempting to convince both himself and the interviewer of his continued good health and the questionability of his diagnosis. Throughout the interview he moved between such efforts at denial and belief that there had been some kind of mistake, contrasted with at other times a very pessimistic and angry acknowledgement of his condition, highlighting how the lived experience of illness may sometimes obstruct the process of acceptance amongst patients.

#### Rationalisation and reappraisal of symptoms

Participants took different steps to help them understand and adjust to their changing health conditions. One such approach could be described as ‘causal theorisation’, whereby patients attempted to relate their symptoms and how they were feeling to medical results, opinions and the status of the disease. This functional understanding helped participants to maintain more positive outlooks, in some cases by supporting a form of denial which enabled them to avoid emotional harm and distress.

The only thing is climbing hills; I do get a bit puffed out. I haven't got the same lung capacity that I had you know the, the radiotherapy could kill off good cells as well as the bad. [Paul, interview 3]

Tom, who throughout the interviews demonstrated a strong interest in medical research, also noted how the enhanced understandings he developed from carrying out his own reading helped to increase his feelings of control over the situation, while it seems ‘learning’ in itself may have been a gratifying and meaningful experience for this participant.

From my point of view you feel you have got some measure of control over what's going on. And even when you can't control it at least you understand why you can't control it you know so it takes the sort of worry factor out of it. [Tom, interview 1]

This embracive approach to medical information was not shared by the majority of patients. A number of patients chose not to engage in the details, explaining that they preferred that the ‘essentials’ were given to them by their health professionals. For example, Jim seemed to view excessive information and learning as a threat, but felt happy with his level of understanding with regards to his condition, treatments and the trial. He described how he practised ‘selective hearing’ and justified this with reference to his wife's experiences. This approach enabled him to maintain a ‘healthy’ level of acceptance, finely balanced between the potential for denial and information ‘overload’.

The thing is if you ask the question they give you the answer. I think the sensible thing is to not give you too much. The worse thing, I mean my wife was told she uh, she was asthmatic she was told, she worried and her asthma got worse or her breathing got worse. [Jim, interview 3]

Patients also prioritised their symptoms and adapted their expectations in order that certain symptoms could be perceived as ‘minor’ and could be ‘lived with’. Jim's focus was on getting his tumour treated and was less concerned with ‘minor’ symptoms such as difficulty swallowing, indicating the high threshold for symptoms and side effects which patients can develop. Several patients also reflected on how their breathing had improved as a result of their chemotherapy, and the following example illustrates how perceived improvements and adjusted expectations can help patients to assess their health positively in spite of their disease. What was important to Tom was not that he got out of breath, which he had come to accept, but that his breath returned more quickly than it used to.

But uh no I'm pretty fine now, obviously I'm still running on one and a bit lungs but uh the important thing is if I do get puffed out (.) uh my breath returns pretty quick now. [Tom, interview 2]

Another strategy used by patients to help them accept and come to terms with their physical condition was through the normalisation of symptoms. Several examples were given of patients finding alternative reasons such as old age and the winter season, to help account for, or at least downplay, more minor symptoms such as tiredness, aches and pains.

Well you know they ask me like ‘if you get tired?’, well you do you know but I mean it's more or less because the winter was here … but I mean I never been like that before so I do say ‘yeah’ I do get more tired than I used to but I mean, ‘cause I'm sixty‐five you [know], don't expect to run a marathon like. [Pete, interview 3]

#### Symptom management and taking control

Participants developed a variety of practical self‐management strategies to help them physically manage some of their symptoms and side effects. Some of these were suggested by health professionals, whilst others came from peers and their own information seeking and ‘trial and error’ processes of investigation. Tom used a throat rinse recommended to him by a fellow clinic patient to manage the discomfort in his throat, while Jim had identified his own ‘fizzy drink’ solution to his swallowing problem. Jim had also learned to alter his breathing techniques following a visit from a health professional, whilst Tom had learned to stop to recover his breath.

You come up with strategies of how to deal with this, how to deal with that you know. Uh same with breathing I mean, I'm not too bad, I can drive around you know uh but you know sometimes I just have to stop, park and get my breath back a bit and then carry on but it's OK I haven't got a problem with it (laughs). [Tom, interview 1]

Within these examples there was a sense of ‘little victories’ as participants drew satisfaction from the steps and strategies which they had mastered and had brought tangible benefits to their physical and emotional wellbeing. This combination of psychological reappraisal and the physical management of symptoms helped to give some participants a sense of control over different aspects of their illness, experience life as more ‘normal’ again and enabled some to ‘feel better’ or even ‘well’ compared with previous times.

Oh I feel it, don't get me wrong I'm a different person. I feel a bit of a fraud, when I look in the mirror I don't look that ‘death in the eyes’ sort of thing if you know what I mean. So uh um there always is, there definitely is one hell of an improvement. [Jim, interview 2]

### Maintaining daily life and relationships

#### Keeping up with hobbies and daily activities

Maintaining daily activities was extremely important for participants’ quality of life, and several patients described being able to keep up with their everyday tasks. Across all three interviews Paul appeared positive as he described being able to carry on with a fairly active life, and also reported to have been effectively treated with radiotherapy.

Yes, I walk to town but I, I do some shopping or go to the library and I catch bus back with the shopping see. I walk my sisters up at (names local site on edge of national park). That's a two mile walk as well. [Paul, interview 2]

Jim, also in apparent good spirits across his three interviews, gave detailed descriptions of his hobbies and commitment to staying as active as possible by regular walking, DIY projects and housework. Such activities helped maintain feelings of normality, control and accomplishment, whilst also avoiding boredom and self‐pity.

I could get a cleaner in, I don't, I like to do it myself, basically because it's a test to see that. I think it is too easy to sit in a chair, I've seen people sit in chairs, um ‘pity me, I'm not well’ and they just fade away, I don't intend to do that, I'll go out with a bang, that's my attitude. I'll go with a mop in my hand or something you know. [Jim, interview 2]

These experiences contrasted heavily with those of Ben who had the appearance of being extremely sick and depressed during both of his interviews. He described how everything had changed for him as he could no longer go to the pub to watch football, go fishing or ‘get round and stuff’.

#### Making adaptations and finding new roles

For many participants, efforts at maintaining daily functioning and quality of life meant making adaptations. Jim had substituted going out for food with going out for teas and coffees due to the difficulties he sometimes experienced eating. He was thus able to keep up with the primary activity of going out with his son at the weekends and maintain the important social aspect of eating. Other participants also described how they gave up some interests, but achieved normality and a sense of purpose through other more attainable activities. Tom described how he had made the decision to avoid pubs during the winter months due to risk of infection, but like Toby and several other participants attached importance to the fact that he was still mobile and able to ‘get around’ and out of the house.

I mean straight after the chemotherapy I tend to stop in uh for a week or so but after that yeah we go out uh shopping or we might have a run out somewhere you know uh I get a bit fed up of being in the house so I do make a conscious effort to go out. [Toby, interview 1]

Tom gave further examples of how simply re‐engaging in previously taken for granted activities, such as eating food and drinking cider, which had been ‘dropped’ during periods of more acute sickness, provided him with a sense of enjoyment and the comforting familiarity of some level of ‘return’ to his way of life.

Um that's something else I'm enjoying now, well not so much beer um (.) I have a couple of pints of uh pear cider at night now (laughs). Yes, yes, things are returning now. [Tom, interview 2]

Tom had also forged a new role for himself in his active engagement with medical research. He was participating in more than one study and had conducted a fair amount of internet‐based research on his condition and treatments. Participating in research provided him with activities to pass the time and help alleviate the boredom which he experienced after having to give up work when he became ill. Tom found being a research participant ‘fun’ and appreciated the attention from research staff. Like several others he also seemed to benefit from an enhanced sense of self‐worth by contributing to research which could help cancer patients in the future.

#### Maintaining social and family life

Social contact and relationships with friends and family were extremely important to participants. Most had spouses who lived with and cared for them, and David and Jim had adult children who had moved back in with them to help out with the house keeping. For other patients who lived alone (Paul) or as a single parent (Mary), the support of siblings was important. Many also described an additional supportive role played by friends and neighbours, illustrating how strong social networks and cohesive communities can function as important resources for patient coping and wellbeing**.**

Jim explained the significance of having close ties with friends and family in terms of providing practical support with shopping and hospital visits, emotional support at difficult times and a sense of purpose and accomplishment in life.

Otherwise you can be demerit yourself ‘I've done nothing with my life, I'm not this, I've not achieved that’ uh we've all got ambitions. I've never achieved an ambition in my life. I've strived but I've never got there. So you suddenly feel well what's the point. But by having these people around you it makes a hell of a difference. [Jim, interview 1]

Some continued to perform supportive care roles for others, such as grandchildren and elderly neighbours, which gave them a reason to stay active and ‘keep going’. Participants also described the important role played by their clinical and research nurses, who were described by one participant as their ‘friend’. These nurses seemed to exceed participants’ expectations in terms of the level of follow up, regular phone contact and the friendly, caring clinical environment, highlighting the potentially therapeutic nature of patient‐professional relationships.

I've had quite a bit of hospital but um this isn't, it's not like hospital really. I know it is, but it's the environment, its, they are not just nurses they're your friend. [Mark, interview 1]

Several patients had also been receiving support from cancer and palliative care charities. They valued the advice and practical support that they gave in terms of accessing grants and communicating with family members, as well as the comfort and reassurance that they gained from talking and sharing their experiences with nurses and fellow patients.

### Confronting the future

#### Active acceptance

Participants responded to their terminal prognosis with varying degrees of acceptance. Whereas some spoke openly about the prospect of dying and the likely limited time that they had left, others barely made reference to this aspect of their illness. Jim in particular seemed to be actively trying to find meaning in and ways of accepting his life situation. At various points in the interviews he made reference to the short amount of time he had left, and was the only participant to mention end of life care as he spoke of his preference for a hospice or hospital death. He seemed to be coming to terms with his terminality with a number of different strategies, which in turn seemed to support feelings of satisfaction and fulfilment. A first of these was through setting himself achievable targets and identifying his priorities and goals. In the following extract Jim demonstrated his acute awareness of having limited ‘time left’ as he talked of hitting ‘six months’ and putting his affairs in order. By viewing his illness as a ‘wake‐up call’ he had identified those things that mattered most to him and was positive about the prospect of enjoying one more summer.

Yeah uh and it's a wakeup call, you suddenly wake up and think ‘well why you doing this?’ What's the point? You know, you've been doing this everyday for 365 days a year for twenty years, why? Oh I won't do it then, I won't do the housework today, I'll leave it. [Jim, interview 3]

Jim also described the importance that he attached to being open about his illness, which not only helped him come to terms with his condition but also enabled his friends and family to ‘pull together’ to support him, in turn enhancing his sense of connectedness and self‐worth. His positive outlook also seemed to be supported by a sense of altruism and the prospect of leaving a positive legacy by participating in medical research (see Harrop *et al*. 2016).

#### Fatalistic acceptance

Some other patients also demonstrated an acceptance of their illness and the uncertainty of their future. In contrast to the acceptance of Jim, which seemed based on a more active appraisal of his life situation, this type of acceptance was linked to the fact that there was little that could be done and therefore no point worrying about it.

You just have to get on with life. Yeah you know it's, you are dealt that hand you just play it you know. [Mark, interview 1]

In the beginning I was depressed, but now I come to terms with it now. I know I got it there and there, there's nothing I can do about it. [Mary, interview 1]

However, such narratives were inconsistently expressed. Mark was only interviewed once due to deterioration in his condition, whilst in the later two interviews Mary had again become depressed and was burdened with worry about how long she had left.

#### *Hope of ‘getting better’; target setting*,* positive action and faith*

A number of patients, including Mark and Mary, seemed to focus more on the prospect of recovery and getting better. Whereas Jim, in negotiating a position of acceptance had set himself short term targets, the target setting which occurred within the talk of these participants was more long term and less realistic. Tom talked about going back to work in six months’ time, whilst Mark identified upcoming milestones in his life as he gave reasons to keep on living.

Yeah, oh well I said to (oncologist) when we first met, 4 years time is our 50th anniversary, the year after that I'm 75 so I've got my free television licence. So that's 5 years and I said we'll round it you to 10, alright? [Mark, interview 1]

Participants also took positive action to try to improve their chances. All participants were receiving cancer treatments and many had joined the clinical trial in the hope of receiving the additional daily injections given to the intervention group. As reported elsewhere these participants described a need to try everything and anything that might help them (Harrop *et al*. 2016). Mary also described at some lengths her efforts to adopt a healthier lifestyle by changing her diet and attempting (unsuccessfully) to give up smoking, which was a source of anxiety and stress across all three interviews. She explained how she was taking these actions for her sons in the hope that this could bring the disease under control.

Even if I don't want to eat it, I've got to do it for my boy. He's only nine … I'm doing it for him I am. I've got a boy [who's] schizophrenic as well, I'm doing it for him. [Mary, interview 1]

Mary also made reference to her faith, spirituality and feelings, which at the time of the first interview were giving her the sense that she was going to get better, just as she had correctly sensed that her father would recover from a terminal condition previously.

But since I've been bad I get these feeling round me all day, I can sense things and I don't know why, it must be because of my illness but I feel like I'm going to get better, don't know why, I might be wrong, I might be. [Mary, interview 1]

In the second or third interviews, however, there was very little reference to faith or instincts and although her treatment had gone well, she reported feeling depressed and seemed far more pessimistic.

#### Depression and hopelessness

Although some patients found ways of talking positively about their future, many also described their worries and anxieties over what lay ahead. Most commonly, and most distressingly, patients described their concerns over leaving family members behind.

The thing is (.) I got cancer on the two lungs [so] there's no hope (.) … The only thing that worries me is (names wife). What's going to happen to her? (tearfully) [David, interview 1]

This concern and distress was understandably most acute for Mary, a single parent with a young child and an adult son with a mental illness. Although her treatment had been going well, she appeared at her most depressed and anxious at the time of her third interview. Talking to her children about her illness had been proving problematic, despite support from a specialist nurse, making it very difficult for her to come to terms with her situation.

Same really I feel depressed all the time. I feel like I'm fed up…I'm worried about my little boy and all; things go through my mind like. [Mary, interview 3]

Ben, who came across as particularly sick and depressed during both of his interviews, also reflected upon the ‘loose ends’ and ‘build up’ of paperwork, which he directly related to his feelings of depression.

## Discussion

This article has identified and given detailed descriptions to some of the ways in which advanced cancer patients respond to and manage different aspects of their illness. As such it adds to the small amount of qualitative research in this area and more specifically helps to address a reported dearth of literature on how lung cancer patients cope with their illness (Ellis *et al*. 2012). By in depth analysis of participants’ talk across different time points three core life concerns are identified in the patient journey, and within these a number of different coping themes which describe participant beliefs and behaviours (see Table 2). While some patient narratives incorporated many of these more positive themes, which in turn seemed to relate to a state of relative wellbeing, their experiences contrasted heavily with those less ‘adjusted’ patients, whose narratives reflected more negative responses, and who seemed less able to pursue some of the coping strategies identified. This article explores the relevance of Antonovosky's concepts of comprehensibility, meaningfulness and manageability for understanding the coping experiences of cancer patients (Antonovsky 1979) and identifies a number of implications for health and social care services.

**Table 2:** Life concerns and coping themes

Core life concern	Coping themes	Relevance to SoC theory
Making sense of and managing one's illness	(Mis)understanding and (non) acceptance of diagnosis	Comprehensibility
	Rationalisation and reappraisal of symptoms	Comprehensibility, Manageability
	Management of symptoms and taking control	Comprehensibility, Manageability
Maintaining daily life and relationships	Keeping up with hobbies and daily activities	Manageability, Meaningfulness
	Making adaptations and finding new roles	Manageability, Meaningfulness
	Maintaining social and family life	Manageability, Meaningfulness
Confronting the future	Active and passive acceptance	Manageability, Meaningfulness
	Hope of getting better: target setting, positive action, and faith	Manageability, Meaningfulness
	Depression and hopelessness	

In the first of these life concerns, ‘making sense of and managing one's illness’, participants used a number of strategies which could be seen to support comprehensibility and manageability. To varying degrees patients took steps to learn about different aspects of their illness and treatments, which included accessing written information and more commonly (and preferably) by speaking with clinicians and other patients, as reported elsewhere (Bulsara *et al*. 2004, Ellis *et al*. 2013, Kvåle and Synnes 2013, Strang and Strang 2001, Taleghani *et al*. 2006). Although some patients described how they practised ‘selective hearing’, it is noted that this does not necessarily imply poor comprehensibility, which is considered optimal when a person understands as much as they want to about a particular situation (Antonovsky 1979, Kvåle and Synnes 2013). In this respect, ‘selective hearing’ and a preference for ‘essential’ information could be seen as strategies which support comprehensibility by enabling patients to avoid information overload and anxieties associated with too much information (Kvåle and Synnes 2013). Just as comprehensibility was constructed by the thoughts and theories of patients with brain tumours in a previous study (Strang and Strang 2001), in this study it was most apparently obstructed for those patients unable to reconcile clinical information with their lived experiences of feeling reasonably healthy. These patients struggled to comprehend, and seemed at times to be in denial of the existence of their illness; a coping strategy also observed in previous research with cancer patients. (Ellis *et al*. 2013, Link *et al*. 2005, Taleghani *et al*. 2006)

By pursuing cognitive and practical strategies aimed at the rationalisation, reappraisal and management of their symptoms, participants not only enhanced their levels of understandings of their illness; they also enhanced their perceived manageability over their illness situations (Bulsara *et al*. 2004, Ellis *et al*. 2013, Strang and Strang 2001). Such strategies included causal theorisation, prioritisation of symptoms, adapted expectations and the normalisation of symptoms. These strategies could be seen to support feelings of control, normality and hope; three constructs which appear to be core to ‘manageability’ for this patient group, and have been widely identified as important for patient and family coping in a palliative context (Bulsara *et al*. 2004, Ellis *et al*. 2012, Kylmä *et al*. 2009, Lin and Bauer Wu 2004, Milberg and Strang 2007, Olsman *et al*. 2014, Strang and Strang 2001). In the literature on chronic illness too, both the processes of normalisation, and the active role of talk in accomplishing this are highlighted (Bury 1982, 2001, Williams 1984). This literature usefully distinguishes two different processes of normalisation in terms of the minimisation and denial of symptoms, as highlighted above, as well as processes whereby the illness is incorporated into adapted lifestyles and is discussed openly with others (Bury 2001). This construction of a ‘new normal’ was also evident in some of our participant narratives, as discussed below.

In the second of the core life concerns ‘maintaining daily life and relationships’, participant efforts could be seen to further support manageability and in particular meaningfulness. Keeping up with hobbies and daily activities, making adaptations and finding new roles, and maintaining social ties and (as far as possible) existing social roles, again facilitated ‘manageability’ by helping participants to maintain a sense of normality (both ‘old’ and ‘new’) and with this feelings of control. These social networks also provided practical and emotional forms of support, the importance of which is widely highlighted in the literature (Bulsara *et al*. 2004, Ellis *et al*. 2012, Kvåle and Synnes 2013, Link *et al*. 2005, Lin and Bauer Wu 2004, Milberg and Strang 2007, Strang and Strang 2001, Taleghani *et al*. 2006). More importantly perhaps, these family and social relationships were a critical source of meaning and motivation in participants’ lives (Kvåle and Synnes 2013, Lin and Bauer Wu 2004, Strang and Strang 2001), and in one case in particular an enhanced sense of connectedness was developed through the illness experience. Likewise, the enjoyment, sense of purpose and comforting familiarity and reaffirming of ways of life enabled by hobbies and daily activities, could also be seen to provide meaningfulness and stability in the changed and changing life situations of these participants (Kvåle and Synnes 2013, Milberg and Strang 2003, Strang and Strang 2001).

As participants ‘confronted the future’ four main types of responses were observed; active and fatalistic acceptance, hope of getting better and depression and hopelessness. Within these narratives a number of different strategies were demonstrated which could be seen to support manageability and meaningfulness. Hope of getting better was supported by accessing medical treatments and undertaking lifestyle changes. In some respects these commitments enhanced ‘manageability’ for patients by providing a perceived chance of improvement and allowing a sense of agency and control. However, they could also prove a source of frustration and anxiety if not fulfilled, and point to the sometimes negative impacts of broader cultural narratives which incline patients with advanced cancer towards staying positive and ‘trying everything’ (see also Harrop *et al*. 2016). By more passive acceptance and belief in fate and higher forces some participants also seemed able to find hope and a reason to ‘not worry’ (Bulsara *et al*. 2004, Ellis *et al*. 2012, Lin and Bauer Wu 2004, Link *et al*. 2005, Taleghani *et al*. 2006). However, such narratives proved difficult to sustain over time and were countered in talk of depression and hopelessness, which as identified elsewhere, undermines perceived manageability (Strang and Strang 2001). For the one participant who seemed to be actively finding ways of coming to terms with his time limited life situation, getting his affairs in order, open communication and ‘pulling together’ within the family facilitated a sense of control and the ‘new’ normalising of the situation (Kvåle and Synnes 2013). For this participant, meaning, hope and a sense of fulfilment was also clearly derived from these strengthened relationships (Strang and Strang 2001; Kvåle and Synnes 2013; Lin and Bauer Wu 2004), by partaking in the activities and interests which he had prioritised (Strang and Strang 2001), and the perceived positive legacy he was leaving by participating in medical research (see also Harrop *et al*. 2016)**.** Parallels can again be drawn here with the chronic illness literature which also highlights the ‘revaluing’ of life that can occur (Bury 2001, Charmaz 1991). However, in the context of advanced disease these processes should be seen not just in terms of rescuing ‘valued life against the onslaught of symptoms’ (Bury 2001: 15), but also negotiating the impending probability of death, suggesting likely differences in the meaning making and prioritisation which may take place.

A number of coping resources are evident, with corresponding implications for health and social care services. Following transactional coping models (Lazarus and Folkman 1984) and Antonovsky's (1979; 1987) conceptualisation of Generalised Resistance Resources, these can be grouped into internal/ individual level resources and social/external resources. Knowledge was one important individual level resource; although some patients favoured less information, others valued information and knowledge as a way of enhancing their feelings of control and ability to cope with their illness (Ellis *et al*. 2012, Kvåle and Synnes 2013, Strang and Strang 2001). These findings highlight the role and value of different types of information and guidance, from both professionals and fellow patients, and the importance of access to the right level or type of information for each individual participant (Kvåle and Synnes 2013). Religion and faith were also important for some patients, helping them at times to maintain hope and acceptance, indicating the need for appropriate psycho‐spiritual support in palliative and end of life care services (Lin and Bauer Wu 2004, Link *et al*. 2005, Taleghani *et al*. 2006).

At an external level, support from family and friends was valued for practical, psychological and emotional reasons (Ellis *et al*. 2012, Strang and Strang 2001). Healthcare professionals were also an important source of support; participants valued the frequent contact from nurses, the friendly clinical environment and the reassurance and emotional support that nurses gave. The significance of this social and emotional context of care has been similarly highlighted in the wider advanced cancer and palliative care literature, with its emphasis on social networks, emotional and psychosocial needs, and on the therapeutic nature of patient‐professional relationships (Nelson *et al*. 2013, Sampson *et al*. 2014). This research thus further demonstrates the need for professional approaches and services which facilitate family communication, provide opportunities for peer support, and simply give patients the time and encouragement to talk not just about their illness but also about their everyday life, interests and families. This kind of communicative approach was not only preferred by participants in a recent study, but also enabled them to find meaning and reasons to live for, in spite of their new life situations and uncertain futures (Kvåle and Synnes 2013).

A limiting factor in this study was the lower number of participants recruited than intended due to the closure of the trial, and the high rate of attrition common to research involving this patient group. However, the richness of the data, and the idiographic approach to analysis which favours small sample sizes, meant that the research generated detailed insights into individual coping experiences, which have theoretical if not empirical generalisability (Smith and Osborn 2003). It does mean, though, that we only captured longitudinally the experiences of those well enough to speak with us and would have missed at follow up those patients who experienced the most severe decline in their health and functionality, along with other changes to their quality of life and psycho‐spiritual wellbeing. The inclusion of only one female participant also means that the reported experiences may not represent core aspects of female coping experiences and should be investigated in future qualitative studies. It is also important to acknowledge that in talking about their illness experiences these participants were engaging in a particular form of psycho‐social coping. Patients facing serious illness experience significant ‘biographical disruption’ to their lives and identities, and through processes of talk and moral accounting attempt to reconstruct their social identities and life narratives (Bury 1982, Williams 1984). When using patient narratives to gain insight into ‘experiences’ of coping and living with illness it is therefore also important to be mindful of the verbal accounting that takes place in such talk (Bury 2001).

## Conclusion

This article has highlighted the complexity and variability in the coping experiences of advanced cancer patients and the need for contextualised and holistic approaches for understanding these experiences. Antonovsky's (1979) theory of Sense of Coherence provides a useful theoretical model for interpreting qualitative data on coping and wellbeing, at least for patient groups still fit enough to participate in research interviews. The three constructs of comprehensibility, manageability and meaningfulness can be applied to help explain how different types of coping responses can support positive adjustment amongst people living with advanced illnesses, with practical implications for service delivery and the inter‐personal and communicative approaches of health and social care professionals.

## Summary of interview topics

**Table:**

Interview One: Topics	Interviews Two and Three: Topics
**Joining the trial** Reasons for joining the trial Understandings of trial purpose, equipoise and different trial arms Preferences for, and responses to trial arm allocation Understandings and views on randomisation Experience of receiving and accessing information on the trial **Participating in the trial** IG‐Experiences of daily injections (administering injections, side effects, support, continuation) CG‐views on daily injections Experience of attending clinics Experience and views on data collection processes Perceived benefits or disadvantages of being on the trial and suggestions for improvement **Treatment experiences and quality of life** Length of time receiving treatment (eg chemotherapy) Understandings of treatment(s) Responses to treatment(s) (side effects and symptom management) Accessing information and support Impact of illness and treatments on quality of life (daily activities, hobbies, social and family life	**Symptom burden, management and quality of life** Symptoms experienced Symptom management and coping Accessing support and information Impacts on quality of life Responses to chemotherapy/ other treatment Experiences of blood clots **Experiences of injecting (IG)** Administering injections and adherence Side effects and effects on daily life Views on continuing with/stopping injections. **Other healthcare and trial experiences** Experience of receiving information on treatment and illness progression Experiences of visiting clinic and accessing healthcare support. Contact with research nurse and experiences of trial related appointments and information. **End of study reflections (Interview 3)** Views on trial arm status now (compared with at start of trial.) Perceived changes in symptoms and side effects over course of illness. Differences between expectations of, and experiences of treatment. General reflections on experiences of participating in the trial and views on participating in medical research.
